# Transthoracic echocardiography vs. cardiac magnetic resonance imaging for the diagnosis of left ventricular thrombus: an updated systematic review and diagnostic accuracy meta-analysis

**DOI:** 10.1093/ehjimp/qyag150

**Published:** 2026-09-19

**Authors:** Ernesto Calderón Martinez, Carla Isabella Miret Durazo, Ajay Kumar, Rafael Pinto-Colmenarez, Palvi Mroke, Zishan Rahman, Ahmad Hammoud, Imran Baig, Lakshmi Sree Pugalenthi, Gabriela Flores Monar, Sachin Sapkota, Diego Ramonfaur

**Affiliations:** Department of Internal Medicine, Texas Tech University Health Science Center At El Paso, 2000 Transmountain, El Paso, TX 79912, USA; Department of Surgery, University of California Irvine School of Medicine, 2800 Chapman Avenue, Orange, CA 92868, USA; Faculty of Medicine, Isra University Faculty of Medicine and Allied Medical Sciences, Hala Road, Hyderabad, Sindh 7100, Pakistan; Faculty of Medicine, Universidad de Carabobo, Barbula, Valencia, Carabobo 2001, Venezuela; Faculty of Medicine, Caribbean Medical University, 5600 N River Road, Rosemont, IL 60018, USA; Faculty of Medicine, Caribbean Medical University, 5600 N River Road, Rosemont, IL 60018, USA; Faculty of Medicine, Ilia State University, Kakutsa Cholokashvili Avenue 2/5, Tbilisi 0162, Georgia; Department of Internal Medicine, Houston Methodist West Hospital, 18500 Katy Freeway, TX 77094, USA; Department of Internal Medicine, Mercy Catholic Medical Center, 1500 Lasdowne Avenue, Darby, PA 19023, USA; Department of Internal Medicine, University of Miami/Jackson Health System, 1611 NW 12th Avenue, Miami, FL 33136, USA; Department of Internal Medicine, Texas Tech University Health Science Center At El Paso, 2000 Transmountain, El Paso, TX 79912, USA; Department of Internal Medicine, Cleveland Clinic, 9500 Euclid Avenue, Cleveland, OH 44195, USA

**Keywords:** cardiac magnetic resonance, left ventricular thrombus, transthoracic echocardiography

## Abstract

Left ventricular thrombus (LVT) is a significant complication of myocardial infarction, associated with increased morbidity and mortality because of the risk of systemic embolization and stroke. Early detection of LVT is therefore important for the timely initiation of anticoagulation therapy. This systematic review and meta-analysis compared the diagnostic accuracy of cardiac magnetic resonance (CMR) with transthoracic echocardiography (TTE) for the detection of LVT. Following PRISMA guidelines, PubMed, Cochrane, CNKI, Scopus, LILACS, Web of Science, Google Scholar, EMBASE, and CINAHL were searched. Random-effects meta-analyses were performed in R (v3.4.3), with pooled proportions or area under the curve (AUC) values reported alongside 95% confidence intervals, and heterogeneity assessed using the *I*² statistic. Eighteen studies involving 3478 participants met the inclusion criteria. Most studies contributed to the primary analysis of TTE performance using CMR as the reference standard, while only five studies allowed direct head-to-head comparison using an independent reference standard. In the head-to-head analysis, CMR demonstrated higher sensitivity (70.5%, 95% CI: 0.554-0.822) and specificity (99.2%, 95% CI: 0.983-0.997) for detecting LVT compared with TTE, which showed a sensitivity of 31.8% (95% CI: 0.252-0.391) and specificity of 96.3% (95% CI: 0.929-0.981). The AUC for CMR was 0.985 compared with 0.59 for TTE. Heterogeneity was significant for TTE (*I*² = 75.8%) but low for CMR (*I*² = 0.0%). Funnel-plot assessment suggested a low likelihood of publication bias. These findings suggest that CMR may provide a diagnostic advantage over TTE for LVT detection, particularly in high-risk patients or when TTE findings are negative or inconclusive. However, this apparent advantage should be interpreted cautiously because most included studies used CMR, particularly late gadolinium enhancement CMR, as the reference standard rather than an independent imaging modality or pathological confirmation, and many studies were historical and used heterogeneous or non-optimized echocardiographic protocols. Further contemporary studies using independent validation standards are needed.

**Systematic Review Registration**: PROSPERO CRD 42024558739

## Graphical Abstract

**Graphical Abstract:**
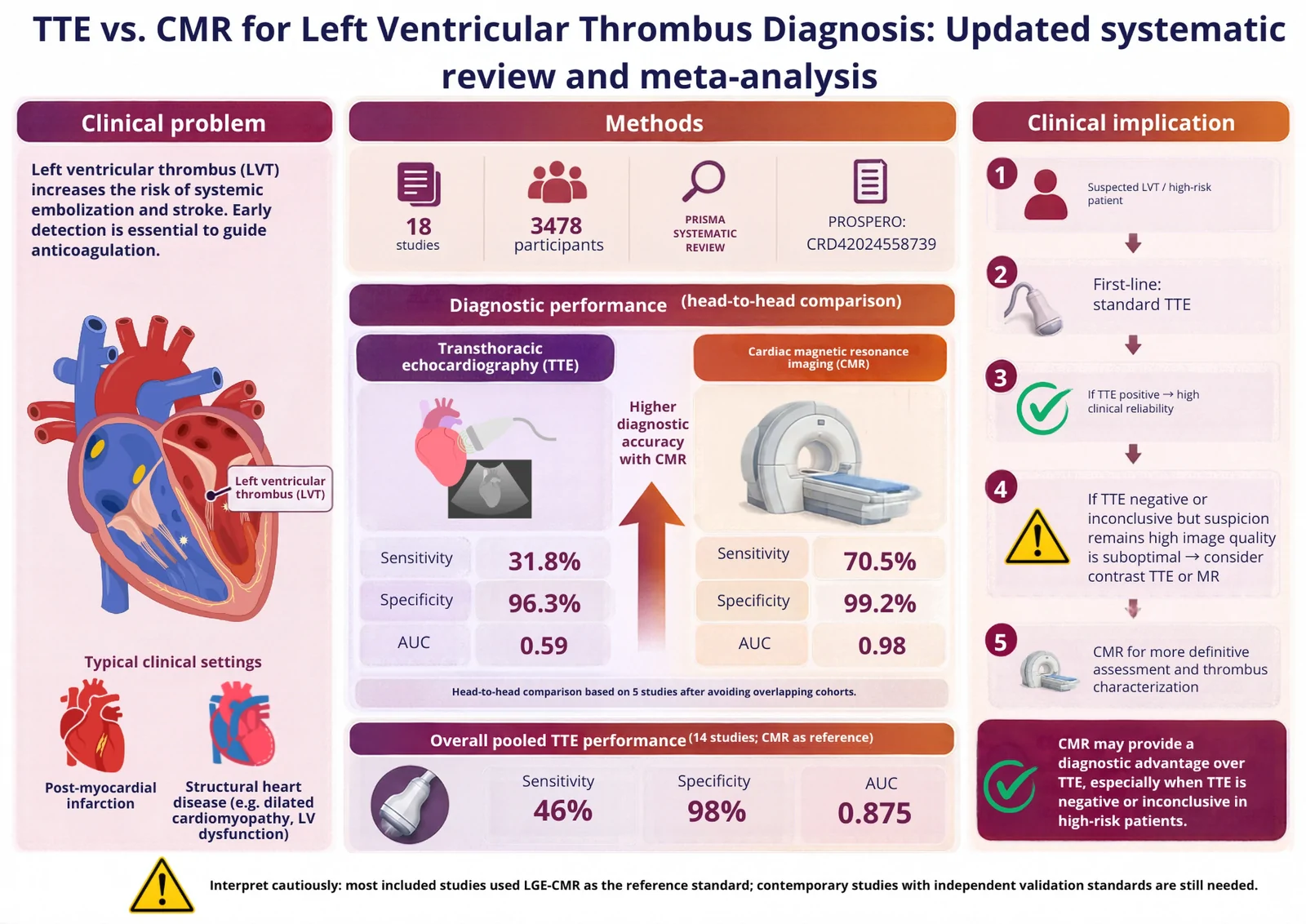

## Introduction

Left ventricular thrombus (LVT) is the formation of a fibrin network or clot comprised of aggregated and contracted platelets, disrupting appropriate blood flow, specifically through the left ventricle.^1,2^ LVT occurs across a broad spectrum of cardiac conditions, including ischaemic cardiomyopathy following myocardial infarction (MI), non-ischaemic cardiomyopathy, heart failure with reduced ejection fraction, and structural heart diseases associated with left ventricular (LV) dilation or regional wall motion abnormalities.^1,3,4^ Regardless of aetiology, LVT is clinically significant due to its potential for systemic embolization, stroke, and major adverse cardiovascular events (MACEs) and is associated with higher morbidity and mortality.^2,3,5^ Depending on thrombus morphology and timing of detection, LVT has been associated with up to a 22% risk of embolization and a 37% risk of MACEs.^2,3,5,6^ Key risk factors for LVT formation include LV apical involvement, ventricular akinesis or dyskinesis, reduced LV ejection fraction (LVEF), and large infarct size; however, these predictors apply across both ischaemic and non-ischaemic substrates.^3,7^ Early detection of LVT is essential to enable timely anticoagulation and reduce embolic risk. For decades, transthoracic echocardiography (TTE) has been the primary imaging tool for LVT assessment, given its wide availability, low cost, and portability. The introduction of echocardiographic contrast agents has improved detection rates, yet TTE remains limited by operator variability, acoustic window quality, and reduced sensitivity for small mural or apical thrombi.^5,8^ Cardiac magnetic resonance (CMR) imaging, particularly late gadolinium enhancement CMR (LGE-CMR), offers superior spatial resolution and tissue characterization, making it a more sensitive modality for LVT detection across diverse clinical populations.^7^

Two prior systematic reviews have addressed this question. Roifman *et al.* (2015) established the diagnostic superiority of LGE-CMR over TTE but included conference abstracts alongside full-text studies, introducing risks of incomplete methodological reporting, data duplication, and unverifiable methods.^5^ Phuah *et al.* (2023) further stratified TTE modalities but did not provide a direct head-to-head diagnostic accuracy comparison between TTE and CMR and similarly included abstracts with inconsistent data reporting and potential double-counting across overlapping cohorts.^9^ Neither review explicitly separated studies in which CMR served as the reference standard from true head-to-head comparisons against an independent gold standard, a distinction that has direct implications for the interpretation of CMR’s apparent diagnostic advantage. The present meta-analysis addresses these limitations by restricting inclusion to full-text peer-reviewed studies, pre-specifying two distinct analytic strata (pooled TTE performance using CMR as the reference and head-to-head analysis comparing CMR and TTE using the reference standards reported in the included studies, which were predominantly based on LGE-CMR), and applying bivariate summary receiver operating characteristic (SROC) modelling to formally compare modality-specific diagnostic accuracy.

## Methods

The present study employed the Preferred Reporting Items for Systematic Reviews and Meta-Analyses (PRISMA) 2020 guidelines to conduct a comprehensive systematic review.^10^ The protocol was registered with the international prospective register of systematic reviews (PROSPERO), in accordance with PRISMA guidelines (CRD 42024558739).^10^

### Eligibility criteria

We included randomized clinical trials, cohort studies, and case-control studies published from database inception through February 2026 in English that evaluated diagnostic strategies for LVT. Eligible studies enrolled participants of any age or sex undergoing diagnostic assessment for suspected or confirmed LVT and provided results for both the index test and the reference standard. The index test was TTE, including standard and contrast-enhanced imaging. Use of echocardiographic contrast was recorded for each study and considered in predefined subgroup analyses. TTE was selected because the primary aim of this review was to determine its diagnostic accuracy for LVT detection. Although contrast-enhanced TTE can improve the definition of the endocardial border and enhance the detection of LVT, we anticipated limited feasibility of a separate pooled ‘contrast-TTE’ diagnostic strategy because (i) relatively few studies used contrast, (ii) contrast was used selectively instead of systematically, and (iii) reporting was not homogenous. Therefore, contrast use was extracted and evaluated descriptively and in subgroup analyses when possible; however, a separate pooled estimate for contrast-enhanced TTE alone was not pre-specified unless sufficient studies utilized contrast consistently. The reference standard was CMR, performed with or without LGE-CMR, with LGE-CMR considered the preferred benchmark given its sensitivity. Studies were required to report sufficient data to extract or calculate complete 2 × 2 contingency tables. The primary outcomes were the sensitivity and specificity of TTE using CMR or LGE-CMR as the reference. In studies where both TTE and CMR were evaluated against an independent gold standard, such as surgical inspection, pathological confirmation, or composite diagnostic criteria, these data were also collected to allow secondary, head-to-head comparisons between modalities. Case series, cross-sectional studies, reviews, protocols, dissertations, conference abstracts, and studies lacking adequate methodological detail were excluded. Variation in the diagnostic reference standard across studies was documented and addressed in predefined subgroup and sensitivity analyses.

### Searching strategy

We searched PubMed MEDLINE, Cochrane, Scopus, LILACS, Web of Science, Google Scholar, clinicaltrials.gov, EMBASE, and CINAHL using the following terms: ‘cardiac magnetic resonance’, ‘echocardiography’, and ‘left ventricular thrombus’ in February 2026. Databases were searched for articles published between the inception of the database and February 2026. For the specific searches per database, refer to our Supplementary material (Supplementary data online, *Table S1*–S9)

### Selection of studies

Title and abstract screening, as well as full-text screening, were conducted independently by two reviewers. Any disagreements were resolved through consensus, with consultation from a third reviewer. Rayyan^11^ was utilized for extracting relevant data during this search.

### Data extraction

Two independent reviewers extracted the data, and disagreements were resolved by consensus. When multiple overlapping reports from the same study were identified, the information from the one containing the most relevant information or the first published report was included. Extracted data included sample sizes, diagnostic test types, and measured outcomes. Conventional methods were employed for data extraction, supplemented by specialized tools such as WebPlotDigitizer (Automeris, Austin, TX, USA) for digitizing data from graphs, Cochrane Calculator for statistical conversions, and StatsToDo for advanced calculations.^12–14^ Extracted outcomes comprised patients detected with thrombus, patients not detected, sensitivity, and specificity. Additional data extracted included subgroup characteristics, such as country of study, sample size, diagnostic test type, and risk-of-bias levels. When multiple publications from the same research group suggested potential overlap in patient populations, only the studies representing clearly distinct cohorts (based on recruitment periods, clinical indications, and patient characteristics) were retained in the head-to-head analysis. Studies from the same group used in the pooled TTE analysis were included only once to avoid double-counting.

### Assessment of risk of bias in included studies

Two independent reviewers evaluated the risk of bias in each study, considering the specific criteria and guidelines provided by the QUADAS tool.^15^ Any reviewer discrepancies have been resolved through discussion with a third, blinded reviewer.

### Statistical analysis

A meta-analysis was performed using the ‘meta’ and ‘mada’ packages in R version 3.4.3.^16^ For single-modality pooled analyses, sensitivity and specificity were estimated separately using random-effects models.^17^ For the head-to-head comparison between TTE and CMR, bivariate random-effects modelling and SROC curve estimation were applied, as this framework jointly models sensitivity and specificity and is the recommended approach for comparative diagnostic accuracy meta-analyses. Formal likelihood ratio testing was used to assess whether the two modalities differed significantly in both parameters. Whenever the number of studies reporting an outcome of interest was insufficient, only a qualitative analysis of the results was performed. For the head-to-head analysis, we included studies that evaluated both CMR and TTE for LVT detection within the same patient population and reported diagnostic outcomes against the reference standard defined by each study. In most included studies, the reference standard was based on CMR, predominantly LGE-CMR; therefore, this analysis should not be interpreted as validation against a fully independent gold standard.

Effect sizes were expressed as proportions and area under the curve (AUC) with 95% confidence intervals. The *I*^2^ statistic assessed heterogeneity, and the following cut-off values were used for interpretation: <25, 25–50, and >50% were considered small, medium, and large heterogeneity, respectively.^18^ For all outcomes, sensitivity analyses according to the leave-one-out method were performed to determine the influence of individual studies on the overall effect, using diagnostic plots proposed by Viechtbauer and Cheung.^19^ Egger’s regression test examined publication bias when 10 or more reports with the same outcome were available.^20^ Whenever possible, subgroup analyses were performed for primary outcomes.

## Results

### Study selection

Our initial search identified 14 148 potential articles across nine databases and additional registers. After removing 4465 duplicate articles, we conducted a screening based on title and abstract, leading to the exclusion of 9657 articles. Two of the 26 articles sought for retrieval were not retrieved as PDF access was unavailable, leaving 24 articles for eligibility assessment. These remaining articles underwent further eligibility screening, excluding six for various reasons: five articles did not align with the study design outlined in our eligibility criteria, and one did not address the relevant diagnostic index test (see Supplementary data online, *Table S10*). Ultimately, 18 studies were included in this review. This process is summarized in our PRISMA flow chart (*Figure 1*).

**Figure 1:**
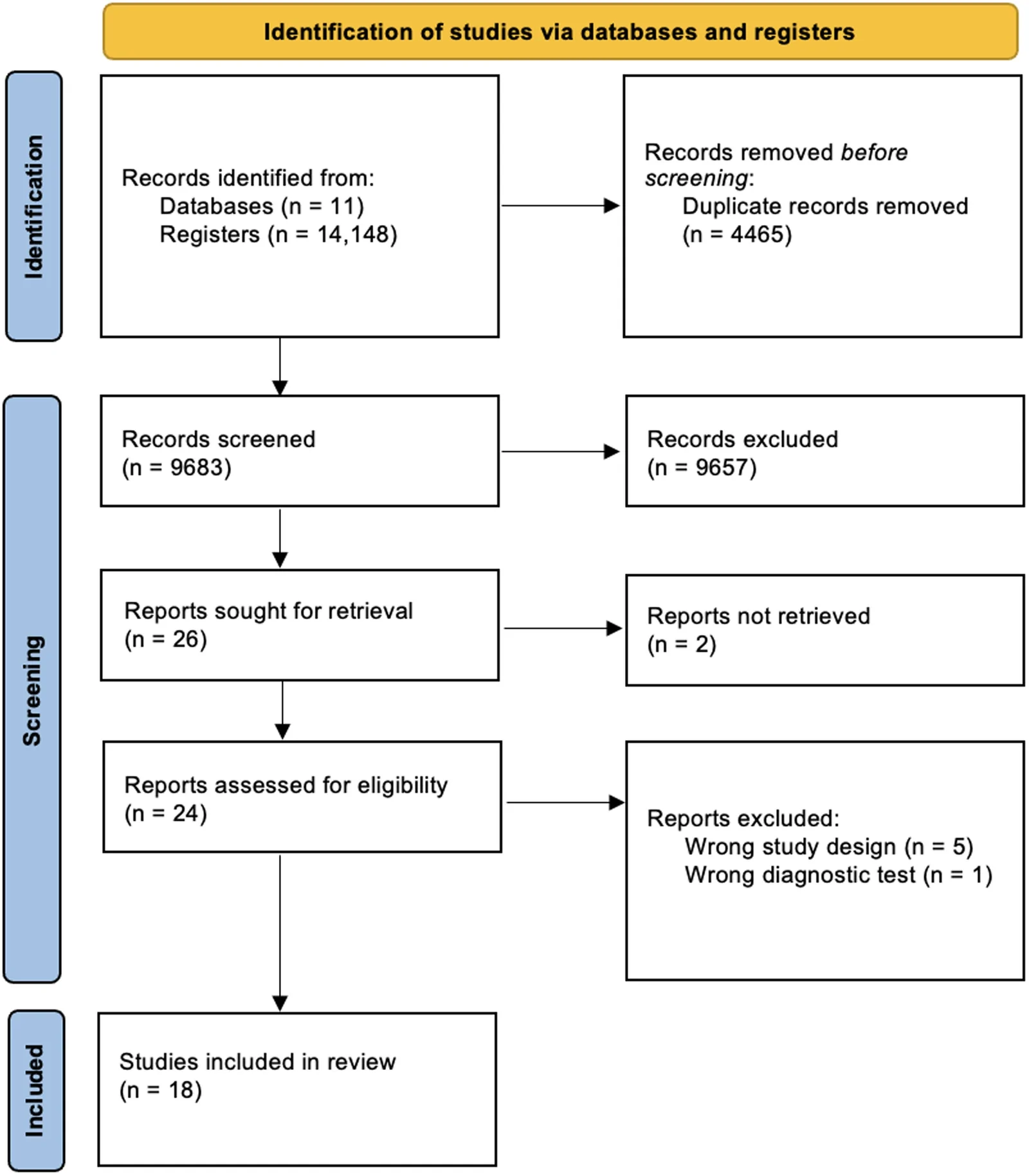
PRISMA flow diagram. PRISMA flow diagram delineates the systematic process of identifying and screening studies across multiple databases, culminating in selecting 18 pertinent studies.

### Characteristics of included studies

The total sample size of the 18 publications presents a total of 3478 participants. The included studies were conducted across a geographically diverse range of countries, including the USA (38.9%), Germany (11.1%), Australia (5.6%), Thailand (5.6%), Kenya (5.6%), Austria (5.6%), the Netherlands (5.6%), France (5.6%), Belgium (5.6%), Canada (5.6%), and Switzerland (5.6%). Among the included studies, 11 (64.7%) evaluated imaging modalities in patients with acute myocardial infarction or ischaemic heart disease. Two studies assessed individuals with left ventricular systolic dysfunction, two studies included patients with known or suspected thrombi, one study included patients with heart failure, one study included patients with structural heart disease and ventricular tachycardia, one focused on patients being considered for implantable cardioverter defibrillator (ICD) implantation, and one evaluated all patients who underwent CMR (see Supplementary data online, *Table S11*). Among the 18 included studies, 11 were prospective in design, seven were retrospective, and only one, Weinsaft *et al.* (2016), was conducted as a randomized controlled trial. Most studies compared 2D TTE against cine-CMR combined with LGE-CMR. In contrast, only a small subset provided data suitable for head-to-head modality comparison using an independent gold standard, with five studies explicitly using LGE-CMR as the gold standard and one study (Srichai *et al.* 2006) using surgery as the definitive reference. These distinctions were preserved during data extraction and analysis. Contrast echocardiography was applied inconsistently across included studies. Only seven studies incorporated contrast in at least a subset of examinations (Velangi 2019; Beavers 2021; Weinsaft 2009, 2011, 2016; Reindl 2023; Surder 2015), and just one study (Beavers 2021) applied contrast systematically to all patients. Across diverse clinical populations—including early and late post-myocardial infarction cohorts, intracardiac thrombus, heart failure, LV systolic dysfunction, coronary artery disease, ICD implantation, and VT ablation—cine + LGE-CMR consistently demonstrated superior sensitivity and reproducibility, while TTE, particularly in non-contrast form, frequently underestimated LVT prevalence or missed small apical or mural thrombi. Therefore, the pooled TTE sensitivity should be interpreted as reflecting predominantly non-contrast routine echocardiography performance in real-world practice, which is known to under-detect small mural or apical thrombi. Anticoagulation regimens varied, ranging from warfarin plus dual anti-platelet therapy or triple therapy to mixed VKA/DOAC/LMWH strategies, aspirin/warfarin, or unreported protocols. The detailed characteristics and findings of these studies are summarized in *Table 1*.^4,21–35^

**Table 1:** General outcomes

Author	Year	Study design	Population	Country	Sample size	TTE protocol	Comparator	Gold standard	Time between studies	Anticoagulation	Key points
Phan J. *et al.*^21^	2019	Prospective	Post-MI (4 days)	Australia	210	TTE 2D	Cine-CMR + LGE-CMR	NA	CMR studies at a median of 4 days (IQR 3–7) Echocardiograms were performed at a median of 4 days (IQR 3–7)	Warfarin + DAPT	CMR examination after acute MI increases the detection rate of LVT formation. Two-dimensional echocardiography without contrast underdiagnoses and significantly underestimates the incidence of LVT compared with CMR.
Velangi P. *et al*.^22^	2019	Retrospective	Intracardiac thrombus	USA	157	TTE 2D with selective contrast	Cine-CMR + LGE-CMR	NA	Within 10 days.	Warfarin-based therapy	Compared with TTE, CMR imaging was more sensitive for the detection of LVT
Barkhausen J. *et al*.^23^	2002	Prospective	Intracardiac thrombus	Germany	24	TTE 2D	Cine-CMR + LGE-CMR	NA	Performed same day	NA	CMR was the most sensitive modality, while contrast-enhanced TTE failed to detect most thrombi.
Beavers D. *et al*.^24^	2021	Retrospective	VT ablation	USA	154	TTE 2D with contrast	Cine-CMR + LGE-CMR	NA	Not reported	Mixed strategy (VKA OR DOACs OR LMWH)	CMR was the imaging modality where most thrombi were identified. CMR should strongly be considered to rule out intracardiac thrombi in patients with aneurysms. CMR was the most sensitive modality, while contrast-enhanced TTE failed to detect most thrombi.
Chaosuwannakit N. *et al.*^7^	2021	Retrospective	LVSD	Thailand	206	TTE 2D	Cine-CMR	LGE-CMR	Not reported	Aspirin/warfarin	CMR imaging was more sensitive to left ventricular thrombi identification compared with transthoracic echocardiography.
Karau B. *et al.*^35^	2025	Prospective	Heart failure	Kenya	100	TTE 2D	LGE-CMR	NA	Within 14 days	Multiple types: aspirin, warfarin, DOAC	TTE showed an excellent negative predictive value in excluding the presence of LVT.
Kim J. *et al.*^36^	2017	Prospective	Post-MI (**28** **±** **7 days** after STEMI)	USA	74	TTE 2D	Cine-CMR + LGE-CMR	NA	Within 24 h	NA	This study showed that apical strain ≤50 achieved 100% sensitivity and 54% specificity for detecting CMR-confirmed LVT, outperforming both ejection fraction and wall motion scoring (AUC = 0.83). Additionally, global longitudinal strain (GLS) correlated strongly with infarct size measured by CMR (*r* = 0.66), with GLS values nearly twice as low in patients with the largest infarcts compared with those with smaller ones.
Srichai M. *et al.*^4^	2006	Retrospective	Post-MI (>8 weeks)	USA	361	TTE 2D	Cine-CMR + LGE-CMR	Surgery	Before surgery. Median 6 (IQR: 0–179 days)	NA	Contrast-enhanced MRI provided the highest sensitivity and specificity for LVT when compared with TTE and should be considered in the care of patients at high risk of LVT formation.
Weinsaft J. *et al*.^25^	2009	Prospective	Mixed	USA	121	TTE with selective contrast	Cine-CMR	LGE-CMR	60% had TTE and CMR same day. The rest with difference between studies of 7 days	NA	DE-CMR is useful for LVT detection among at-risk patients. Thrombus was associated with infarct size—an additional parameter provided by DE-CMR. While TTE diagnostic performance was markedly improved by a strategy of routine contrast use, prevalence was lower than by DE-CMR, especially for mural thrombus and small apical thrombus.
Reindl M. *et al.*^26^	2023	Prospective	Post-MI (3 days)	Austria	870	TTE 2D with selective contrast	Cine-CMR + LGE-CMR	NA	CMR performed 3 (IQR 2–5) days and TTE 3 (IQR 2–4) days after primary PCI	NA	CMR is the preferred imaging technique with the highest diagnostic accuracy for LVT. However, TTE is still widely used to diagnose LVT in everyday clinical practice, yet previous studies consistently reported substantial limitations in the diagnostic accuracy of TTE.
Delewi R. *et al.*^27^	2012	Prospective	Post-MI (3–4 days)	Netherlands	200	TTE 2D	Cine-CMR + LGE-CMR	NA	3–4 days after MI. TTE and CMR same day	Triple therapy (VKA + DAPT)	Routine TTE had a low sensitivity for the detection of LVT, and the interobserver variation of TTE was large when compared with CMR.
Meurin P. *et al.*^28^	2015	Prospective	Post-MI (30 days)	France	100	TTE 2D	Cine-CMR + LGE-CMR	NA	30 days after MI. TTE and CMR same day or consecutive days	VKA + DAPT, enoxaparin bridging, duration ≥6 months	When a search for thrombus is pre-specified, the accuracy of TTE is high as compared with DE-CMR.
Mollet N. *et al.*^29^	2002	Prospective	Post-MI	Belgium	57	TTE 2D	Cine-CMR	LGE-CMR	Within 24 h	NA	Non-visualized lesions were most frequently located in LV apex and had a larger size than non-visualized lesions on MRI. This study also suggests a better identification of LVT than with presently used clinical imaging modalities, such as TTE.
Joshi S. *et al.*^30^	2012	Retrospective	ICD implantation	Canada	52	TTE 2D	Cine-CMR + LGE-CMR	NA	Within 30 days	NA	CMR was substantially more reproducible than TTE for EF, and the agreement between techniques was only modest.
Staab W. *et al.*^31^	2013	Retrospective	CAD	Germany	171	TTE 2D	Cine-CMR + LGE-CMR	NA	CMR 18 ± 7 days after echocardiogram	NA	CMR was found to be more accurate than TTE in identifying intracardiac masses in patients with impaired LVEF and CAD.
Surder D. *et al.*^32^	2015	Prospective	Post-MI (6 days)	Switzerland	177	TTE 2D with selective contrast	Cine-CMR + LGE-CMR	NA	Within 24 h	Oral anticoagulation + DAPT; LMWH/UFH bridging until INR 2–3	CMR is the ideal test for LVT detection; however, TTE is adequate in the absence of additional testing.
Weinsaft J. *et al.*^33^	2016	Prospective RCT	Post-MI	USA	201	TTE 2D with selective contrast	Cine-CMR	LGE-CMR	Within 7 days	DAPT	DE-CMR yielded improved overall thrombus detection; however, apical wall motion on a non-contrast TTE can be an effective stratification tool to identify patients in whom DE-CMR thrombus assessment is most warranted.
Weinsaft J. *et al.*^34^	2011	Retrospective	LVSD	USA	243	TTE with selective contrast	Cine-CMR	LGE-CMR	30 days post-MI. TTE and CMR within 24 h	NA	TTE is inferior to CMR in detecting the presence of LVT formation. CMR was overall more accurate, sensitive, and specific in detecting thrombus formation.

CAD, coronary artery disease; CMR, cardiovascular magnetic resonance; DE-CMR, delayed-enhancement cardiovascular magnetic resonance; EF, ejection fraction; ICD, implantable cardioverter defibrillator; LV, left ventricle; LVEF, left ventricle ejection fraction; LVSD, left ventricular systolic dysfunction; LGE-CMR, late gadolinium enhancement cardiovascular magnetic resonance; LVT, left ventricle thrombus; MI, myocardial infarction; MRI, magnetic resonance imaging; RCT, randomized controlled trial; TTE, transthoracic echocardiography.

*Values without contrast were extracted for analysis.

### Risk of bias within studies

Risk of bias was assessed using the QUADAS-2^15^ tool across four domains (patient selection, index test, reference standard, and flow and timing). Overall, 76% of studies were judged at low risk of bias, 18% had unclear risk due to insufficient reporting, and 6% were considered high risk (*Figure 2*). Most studies demonstrated adequate methodology in patient selection, index test conduct, and reference standards, supporting the robustness of the pooled estimates. However, incomplete reporting in some studies may limit full confidence in the precision of the diagnostic accuracy results.

**Figure 2:**
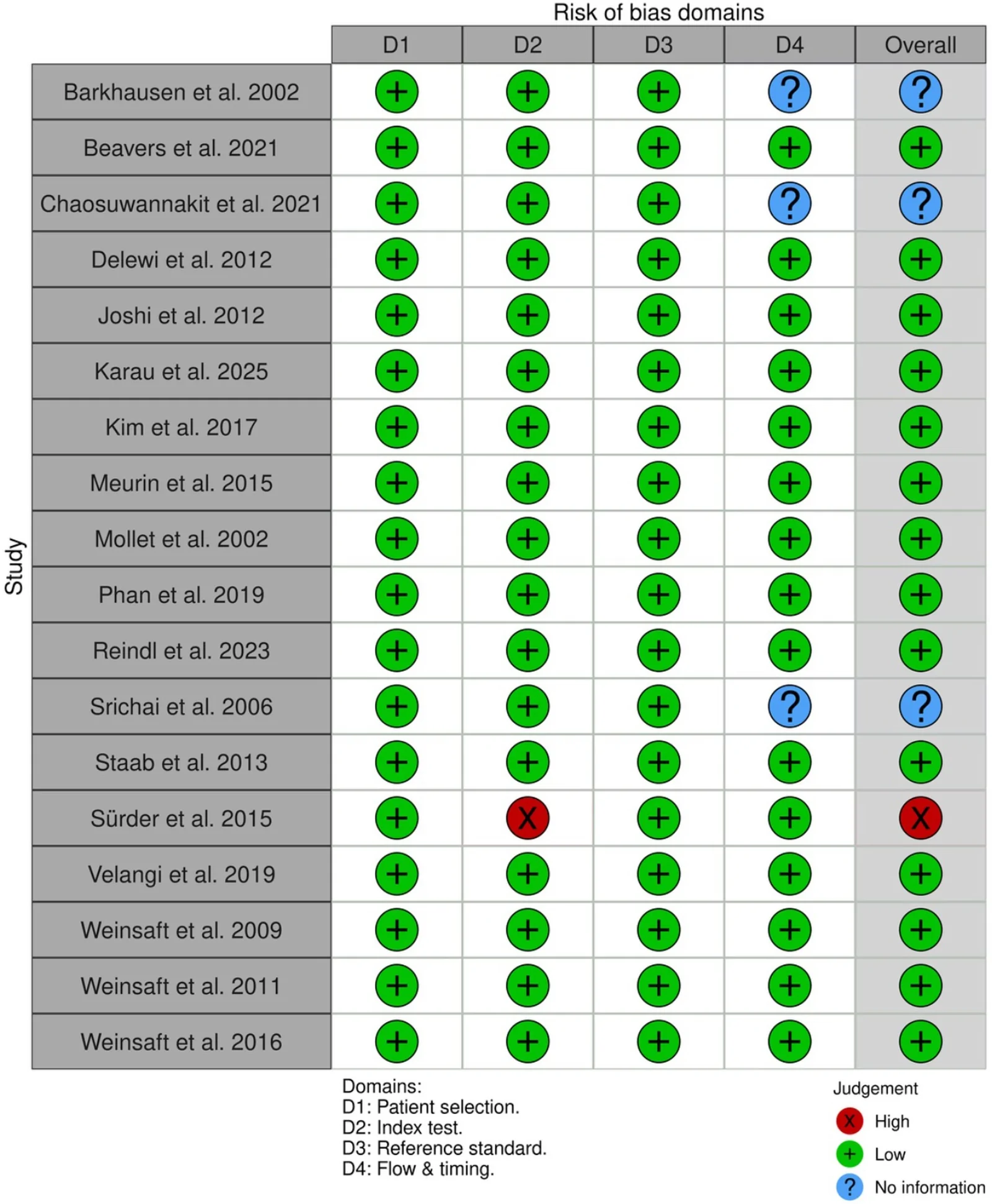
Risk of bias. Risk of bias of studies included in the study. A total of 76% of included studies in the analysis reported low risk of bias, 18% reported no information, and 6% reported high risk of bias.

### Diagnostic accuracy of TTE

In the evaluation of TTE for detecting thrombus, CMR was utilized as the reference standard. The univariate sensitivity analysis included 14 studies with 348 observations. The random-effects model estimated the sensitivity at 0.46 (95% CI: 0.33 to 0.60; *I*^2^ = 75.6%). Because most included cohorts used routine (predominantly non-contrast) TTE, these pooled estimates are best interpreted as the diagnostic performance of standard clinical TTE. The test of heterogeneity showed significant variability among studies (*Q* = 53.36, *P* < 0.01; *Figure 3A*). Specificity analysis was based on 14 studies with 2570 observations. The random-effects model estimated the specificity at 0.98 (95% CI: 0.97 to 0.99, *I*^2^ = 65.4%; *Figure 3B*). A bivariate analysis using the SROC curve yielded an AUC of 0.875 (95% CI: 0.659 to 0.954), indicating good overall diagnostic performance of TTE in detecting thrombus (*Figure 3C*). Threshold analysis indicated that the threshold effect was insignificant (correlation coefficient = −0.007). The pooled diagnostic odds ratio (DOR) for TTE was 43.4 (95% CI, 17.9–105.0, *I*^2^ = 71.3%), indicating high discriminatory ability.

**Figure 3:**
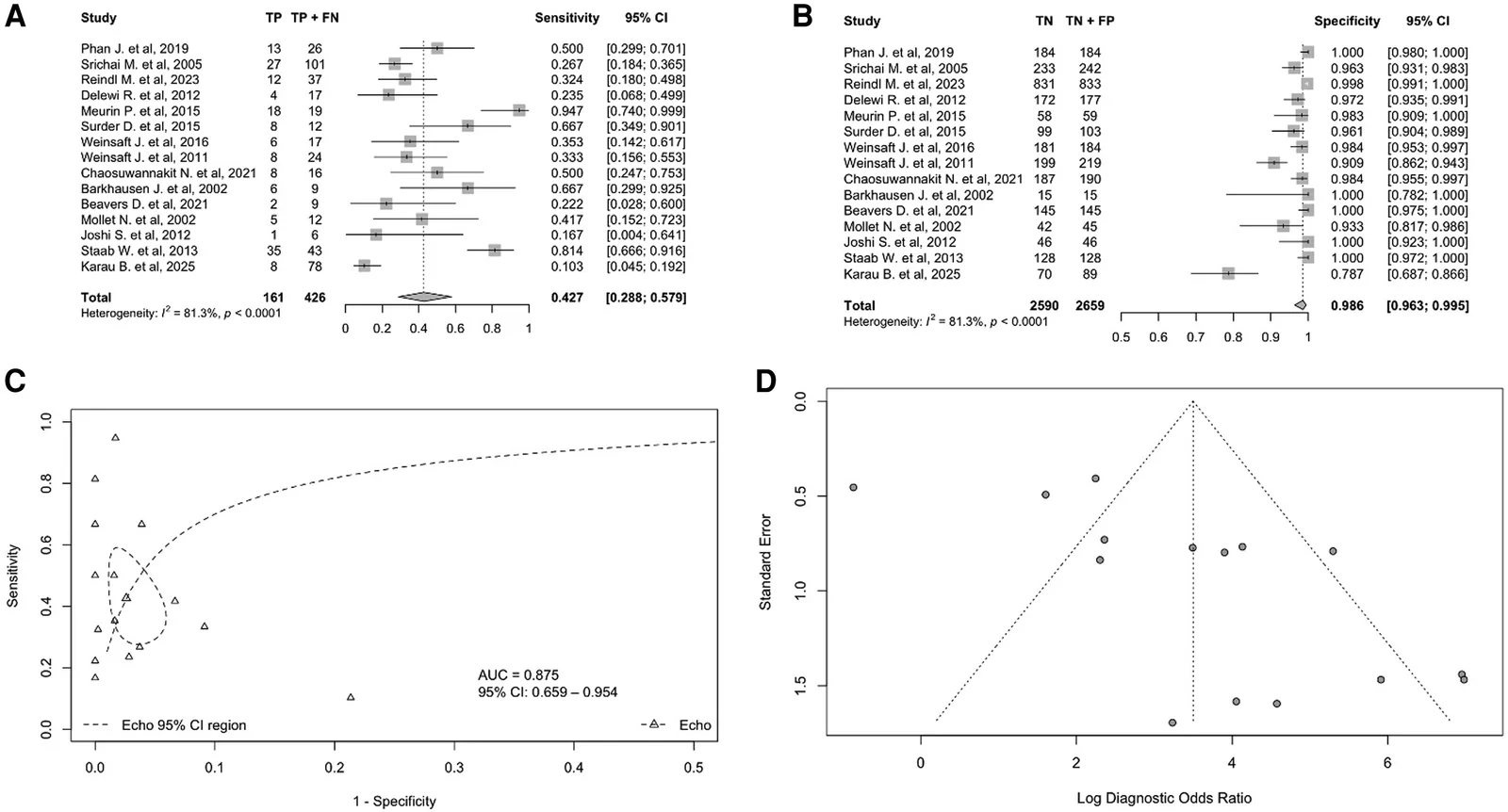
(*A*) Forest plot showing sensitivity accuracy of TTE for detecting thrombus. (*B*) Forest plot showing specificity accuracy of TTE for detecting thrombus. (*C*) SROC curve of TTE for detecting thrombus. (*D*) Funnel plot assessing publication bias among the included studies.

#### Subgroup analysis and sensitivity analysis

For sensitivity, the subgroup analysis did not identify differences for study design or contrast used in the TTE (*P* > 0.05 for both) but identified differences for the indication of evaluation (*P* < 0.01; Supplementary data online, *Table S12*), with several single-article subgroups. The subgroup of studies with post-myocardial infarction indication reveals one of the strongest sensitivity values, with high heterogeneity: 0.46 (95% CI: 0.28 to 0.64, *I*^2^ = 69.6%). The leave-one-out analysis for sensitivity identified the article by Staab *et al.* (2013) as influencing the overall effect size and heterogeneity (see Supplementary data online, *Table S13*); an analysis excluding these articles led to reduced and moderate heterogeneity and modified sensitivity of 0.42 (95% CI: 0.31 to 0.56, *I*^2^ = 58.9%). For specificity, neither indication, study design, nor use of contrast in TTE identified differences (*P* > 0.05 for all; Supplementary data online, *Table S14*). Leave-one-out analysis for specificity identified Reindl M. *et al.* (2023) as influencing the overall effect size and heterogeneity; an analysis excluding these articles led to a reduced and null heterogeneity and modified specificity of 0.98 (95% CI: 0.96 to 0.99, *I*^2^ = 39.8%; Supplementary data online, *Table S15*).

### Publication bias

Although visual inspection of the funnel plot of the log DOR suggested minor asymmetry, Deeks’ funnel plot asymmetry test demonstrated no evidence of publication bias (*t* = 0.04, *P* = 0.97). Most studies cluster near the centre with low standard errors (SE), suggesting high precision in their estimates of DOR. However, a few studies, such as those by Meurin P. *et al.* (2015) and Staab W. *et al.* (2013), exhibit higher SEs and more extreme DOR values, reflecting lower precision and potential variability in diagnostic performance (*Figure 3D*).

### TTE vs. CMR

All studies included in the head-to-head analysis required that each enrolled patient underwent both TTE and the reference standard examination within the same study. Only seven studies met the criteria for a true head-to-head comparison between TTE and cine-CMR, using LGE-CMR as the gold standard, and Srichai et al. (2006), using surgery as the gold standard. However, four of these were produced by the same research group (Kim *et al.* 2017; Weinsaft 2009; Weinsaft 2011; Weinsaft 2016), raising concerns about overlapping patient populations. To avoid double-counting, only Weinsaft (2011) and Weinsaft (2016) were retained in the analysis, as these two studies clearly represent distinct cohorts separated by different recruitment periods, clinical indications, and disease characteristics. We analysed sensitivity and specificity across multiple studies. For TTE, the sensitivity was derived from five studies involving 170 observations and 54 events, yielding a sensitivity proportion of 0.31 (95% CI: 0.25 to 0.39, *I*^2^ = 4.0%; *Figure 4A*). In contrast, CMR’s sensitivity, based on five studies with 141 observations and 99 events, was significantly higher at 0.70 (95% CI: 0.55 to 0.82, *I*^2^ = 66.4%; *Figure 4C*). Specificity for TTE, calculated from five studies with 880 observations and 842 events, was 0.96 (95% CI: 0.92 to 0.98, *I*^2^ = 75.8%; *Figure 4B*). For CMR, specificity from five studies with 790 observations and 784 events was 0.99 (95% CI: 0.98 to 0.99, *I*^2^ = 0.0%; *Figure 4D*). Bivariate modelling showed that TTE had limited diagnostic performance (AUC 0.59; 95% CI, 0.32–0.95), whereas CMR demonstrated substantially greater accuracy (AUC 0.98; 95% CI, 0.79–0.98), with a statistically significant AUC difference of 0.39 (95% CI, 0.00–0.65; *P* = 0.04; *Figure 4E*). Consistently, the pooled DOR for TTE was modest (13.9; 95% CI, 5.75–33.70; *I*^2^ = 50%), while CMR achieved a markedly higher DOR of 281.9 (95% CI, 112.79–704.66; *I*^2^ = 0%). Likelihood ratio tests demonstrated that forcing the two modalities to share the same sensitivity significantly worsened model fit (χ^2^ = 9.18; *P* = 0.002), and the same occurred when specificity was constrained to be equal (χ^2^ = 7.62; *P* = 0.006). These results indicate that TTE and CMR differ significantly in both sensitivity and specificity, further supporting the diagnostic advantage of CMR and the need for separate test-specific SROC estimates. The head-to-head analysis is based on a markedly smaller evidence cohort than the overall number of included studies (five studies after overlap), and the pooled estimates should be interpreted as less precise and more sensitive to single-study influence than estimates derived from the full 18-study dataset.

**Figure 4:**
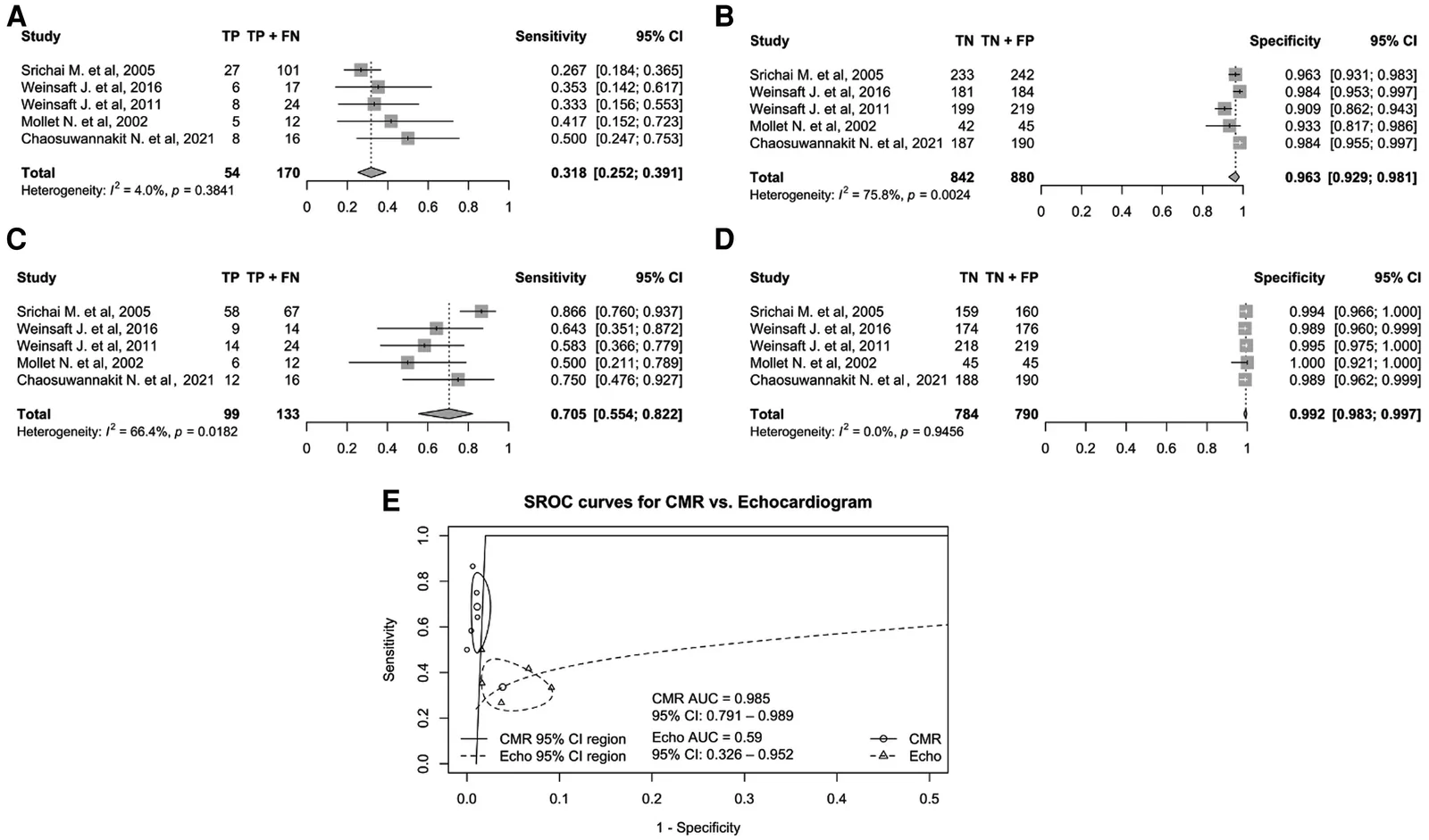
(*A*) Forest plot showing sensitivity accuracy of TTE for detecting thrombus. (*B*) Forest plot showing specificity accuracy of TTE for detecting thrombus. (*C*) Forest plot showing sensitivity accuracy of CMR for detecting thrombus. (*D*) Forest plot showing specificity accuracy of CMR for detecting thrombus. (*E*) SROC curve of TTE compared with CMR for detecting thrombus.

#### Subgroup and sensitivity analysis

Subgroup analysis for specificity values of TTE compared with the gold standard by study design and use of contrast in echo did not reveal any statistically significant difference (*P* > 0.05 for both), but the indication of screening of post-myocardial infarction showed a specificity of 0.97 (95% CI: 0.95 to 0.98, *I*^2^ = 37.5%; Supplementary data online, *Table S16*). Leave-one-out analysis identified three of the five articles as influencing the outcome; an analysis excluding these articles was not performed due to the low number of studies remaining (see Supplementary data online, *Table S17*). For the sensitivity values of CMR, the subgroup analysis did not identify differences for study design, indication, or use of contrast in the TTE (*P* > 0.05 for all; Supplementary data online, *Table S18*). The leave-one-out analysis identified three of the five articles as influencing the outcome; an analysis excluding these articles was not performed due to the low number of studies remaining (see Supplementary data online, *Table S19*).

### Publication bias

The funnel plot for TTE shows the relationship between the SE and the DOR for the studies included. The funnels demonstrate asymmetrical distribution around the overall estimate, indicating a possible likelihood of publication bias. Deeks’ test cannot be performed due to the low number of studies (see Supplementary data online, *Figure S1*). The funnel plot for CMR illustrates a symmetrical and narrower distribution of studies around a higher log DOR; Deeks’ test cannot be performed due to the low number of studies (see Supplementary data online, *F*igure  *S2*).

## Discussion

This study evaluates the diagnostic accuracy of TTE for detecting LVT compared with CMR. Critically, CMR’s apparent diagnostic advantage must be interpreted with an important caveat: in the majority of included studies, CMR served as the reference standard against which TTE was evaluated. This design, inherent to the available literature rather than a choice of the present authors, means that CMR performance estimates reflect its role as the benchmark, not an independent validation of its accuracy against a pathological or surgical gold standard. The head-to-head analysis using an independent reference standard, while limited to five studies, provides a more methodologically rigorous comparison between the two modalities. Compared with prior systematic reviews, including those by Roifman *et al.* and Phuah *et al.*, the present analysis includes a larger number of studies, a substantially expanded patient population, greater methodological rigour, and more recent CMR techniques. Importantly, we explicitly separated pooled analyses in which TTE was evaluated using CMR as the reference standard from true head-to-head comparisons where both TTE and CMR were performed in the same patients. This distinction was not consistently made in earlier meta-analyses and represents an important contribution of the present work. Our qualitative synthesis included 18 studies totalling 3478 participants, with the head-to-head dataset substantially smaller (five studies), providing a more direct comparison between modalities but with lower precision and greater susceptibility to study-level influence. Relatedly, the AUC for TTE in the head-to-head analysis (0.59) is notably lower than the SROC-derived AUC from the pooled analysis (0.875; *Figure 3C*). This discrepancy reflects the methodological differences between the two analytic strata rather than a statistical inconsistency: in the pooled analysis, TTE’s consistently high specificity (∼0.98) across 14 studies elevates the SROC-AUC despite variable sensitivity; in the head-to-head analysis against an independent gold standard, TTE’s low sensitivity (0.318) across five studies drives the AUC downward. These two metrics therefore characterize TTE performance in fundamentally different analytic contexts and are not directly comparable. An important interpretive nuance is that the pooled TTE sensitivity (∼43–46%) represents the diagnostic performance of predominantly non-contrast, non-dedicated echocardiography in routine clinical practice—not the intrinsic diagnostic ceiling of the modality. Evidence consistently demonstrates that sensitivity improves substantially with contrast enhancement (Weinsaft *et al.* 2009; Mansencal *et al.* 2007) and with dedicated LVT-detection protocols including systematic apical window optimization (Meurin *et al.* 2015). This pooled estimate therefore reflects implementation variability across heterogeneous real-world settings and should not be interpreted as the maximum achievable performance of TTE as a modality.

We showed that CMR offers greater sensitivity, specificity, and overall accuracy in detecting LVT compared with TTE. Our study aligns with previous systematic reviews and meta-analyses, which consistently demonstrate the improved diagnostic accuracy of LGE-CMR.^5^ Consequently, LGE-CMR is considered the preferred method for detecting LVT.^5^ Our study includes 18 articles and a substantial cohort of 3478 participants, the largest to date in this field. In contrast, previous systematic reviews by Phuah *et al*.,^9^ which included only seven articles, and Roifman *et al*.,^5^ who analysed 11 articles, used smaller cohorts of 803 and 2113 participants, respectively. The most methodologically comparable prior work, Phuah *et al.* (2023), stratified TTE into non-contrast, contrast-enhanced, and apical wall motion scoring subgroups. However, several limitations constrain the conclusions of that analysis. First, it included conference abstracts alongside peer-reviewed studies, introducing the risk of incomplete methodological reporting and unverifiable data. Secondly, cross-examination of the included cohorts reveals overlapping patient populations across publications from the same research groups. Thirdly, while the separation of contrast and non-contrast TTE is conceptually appealing, the heterogeneity in how contrast was administered across studies (selectively in some, systematically in only one) limits the interpretability of a pooled contrast-TTE estimate. In the present analysis, we extracted and reported contrast use descriptively and evaluated it in pre-specified subgroup analyses; however, a separate pooled estimate for contrast-enhanced TTE was not generated because the conditions necessary for a meaningful pooled estimate were not met: of the seven studies that used contrast in any patients, only Beavers *et al.* (2021) applied it systematically to all participants. In the remaining six, contrast was administered selectively, introducing differential verification bias that would invalidate a pooled contrast-TTE estimate. The pooled TTE estimates here therefore reflect predominantly standard non-contrast echocardiography, which represents real-world clinical practice for the majority of patients worldwide.^9^ Furthermore, the included studies span over two decades (2002–2025), during which echocardiographic technology has evolved substantially. Second-harmonic imaging, high-frequency transducers, and microbubble contrast agents have improved endocardial border definition and LVT detection compared with the fundamental-frequency imaging available in earlier studies. Studies conducted prior to 2010 may therefore underestimate what is achievable with contemporary platforms and systematic contrast protocols. This technological heterogeneity represents an underappreciated source of variability in pooled TTE sensitivity and should be considered when extrapolating the present results to current clinical practice.^5,8^ Additionally, anticoagulation therapy, inconsistently documented across studies, may have reduced thrombus size and echogenicity, further impairing TTE detection and contributing to underestimation of its true diagnostic performance.^37^

The results of our systematic review are significant because they highlight the greater accuracy of LGE-CMR in the clinical detection of LVT. This recommendation must be weighed against known drawbacks, including contraindications (e.g. patients with renal dysfunction with indexed estimated glomerular filtration rate <30 mL/min/1.73 m^2^), increased acquisition cost, availability, and the requirement for patient breath-holding (which can dramatically affect image quality).^38^ Even so, TTE remains a valuable component of clinical practice, particularly in settings where rapid assessment, portability, or contraindications to CMR influence decision-making. A clinically important nuance is that the thrombi most commonly missed by non-contrast TTE (small, flat, mural thrombi) carry substantially lower embolic risk than large, protruding, or mobile thrombi, which are more reliably detected by both modalities. Available evidence indicates that protruding or pedunculated thrombi are associated with significantly higher rates of systemic embolization compared with mural thrombi.^6,27^ The AHA 2022 Scientific Statement on LVT management explicitly recognizes thrombus morphology as a determinant of embolic risk and a factor in anticoagulation decision-making.^6^ Consequently, the clinical impact of CMR’s higher sensitivity should not be interpreted as equivalent across all thrombus subtypes: detection of an additional small mural thrombus by CMR in a patient with a negative TTE does not carry the same management urgency as a missed large protruding thrombus. Future studies integrating thrombus morphology with clinical outcomes are needed to establish the incremental clinical, not merely diagnostic, benefit of CMR over TTE. Based on the present findings, we propose the following stepwise clinical framework. First, standard non-contrast 2D TTE should serve as the first-line assessment in all patients with suspected LVT or high-risk features such as anterior STEMI (ST-Elevation Myocardial Infarction), severely reduced LVEF, or apical wall motion abnormality. A TTE-detected thrombus carries high clinical reliability given TTE’s specificity of 96.3% and warrants anticoagulation initiation without further imaging. Secondly, if TTE image quality is suboptimal, apical visualization is inadequate, or clinical suspicion remains high despite a negative non-contrast TTE, contrast-enhanced TTE should be performed as an intermediate step before CMR referral. Thirdly, CMR should be pursued when TTE—with or without contrast—is inconclusive, when high clinical suspicion persists despite negative imaging, or when precise thrombus characterization is required to guide management. In resource-limited settings where CMR access is constrained, TTE with systematic contrast use represents a reasonable intermediate strategy, with CMR reserved for high-suspicion or diagnostically uncertain cases.^5^ Our findings have significant implications for clinical decision-making. Future research should investigate how myocardial remodelling affects thrombus visualization in heart failure, examine the timing gap between CMR and TTE examinations, and evaluate the incremental value of contrast-enhanced TTE in contemporary practice. When comparing TTE and CMR for diagnosing LVT, cost and accessibility emerge as crucial considerations. CMR scanners carry significantly higher acquisition and operating costs,^27^ and access challenges are particularly pronounced in socioeconomically disadvantaged and rural populations. TTE’s high specificity supports its use as a rapid and cost-effective initial test when clinical suspicion is high, potentially reducing unnecessary CMR referrals, whereas CMR’s greater sensitivity and tissue characterization make it particularly valuable in equivocal or diagnostically uncertain cases. Both modalities face feasibility limitations: TTE may be degraded by obesity or chronic pulmonary disease, while CMR is contraindicated in patients with certain metallic implants or claustrophobia.^39^ Tailoring modality selection to the individual clinical context optimizes both diagnostic accuracy and resource utilization.

Artificial intelligence (AI) may improve LVT detection across both TTE and CMR, potentially by reducing operator dependence, standardizing image acquisition, assessing image quality, optimizing views, and assisting with thrombus recognition. Future studies should develop and externally validate multimodality AI models for LVT detection using contemporary imaging protocols and robust independent reference standards.

### Limitations

This study offers several strengths, including a comprehensive search strategy, rigorous methodological appraisal, and inclusion of diverse imaging protocols reflecting real-world diagnostic practice. Nonetheless, several limitations merit consideration. Inclusion of case-enriched cohorts may introduce spectrum bias, whereby artificially elevated disease prevalence inflates sensitivity estimates. Although QUADAS-2 Domain 1 flagged studies at risk of this bias and subgroup analyses by design yielded no statistically significant differences (*P* > 0.05), residual confounding cannot be excluded. The evidence base relied predominantly on retrospective or non-randomized prospective observational studies, which are more susceptible to selection and workup bias than protocol-driven designs such as that of Weinsaft *et al.* (2016). Most included studies used CMR rather than pathology as the reference standard. Importantly, reported CMR sensitivity and specificity frequently reflect comparisons between different CMR techniques rather than against an independent gold standard, which may overestimate apparent diagnostic performance and limit its interpretation as an absolute benchmark. Clinical factors such as myocardial remodelling in heart failure may further alter thrombus morphology and impair visualization. The wide range of TTE-to-CMR intervals, from same-day assessment to delays exceeding 100 days in Srichai *et al.* (2006), represents a source of interval misclassification bias that could not be formally modelled due to inconsistent reporting, creating potential for false negatives and false positives. The head-to-head dataset was small, limiting precision and making pooled estimates sensitive to single-study effects despite low statistical heterogeneity in some parameters. High statistical heterogeneity was observed across analyses, weakening pooled estimates. Pooled single-modality TTE analyses used univariate random-effects models; bivariate modelling was applied in the head-to-head comparison but could not be extended to the full 18-study dataset due to absent paired sensitivity–specificity data from a common reference standard. *I*^2^ values should be interpreted cautiously, as this statistic has low power to detect true heterogeneity when few studies are included, and low statistical heterogeneity does not imply clinical homogeneity. The primary analyses used separate univariate random-effects models for sensitivity and specificity rather than a hierarchical diagnostic meta-analysis framework, which may provide less robust estimates and does not fully account for the correlation between these measures. Although funnel plots suggested potential publication bias for TTE, the conclusion favouring CMR must be tempered by variability in contrast use, CMR sequence heterogeneity (cine vs. LGE), differing post-infarction imaging intervals, and absence of pathology correlation. Sparse and inconsistent reporting precluded subgroup analyses of contrast-enhanced TTE and other sources of heterogeneity, including imaging timing, image quality, operator expertise, and infarct size. Future research should prioritize prospective studies with consecutive enrolment, standardized imaging timing, pathology correlation where feasible, evaluation of contrast-enhanced TTE, and harmonized diagnostic criteria to enable more robust accuracy assessments.

## Conclusion

Available evidence suggests CMR may have a diagnostic advantage over TTE in detecting LVT, especially in high-risk patients. However, this apparent advantage is constrained by the lack of a robust independent reference standard and by the predominance of historical studies using heterogeneous and often non-optimized echocardiographic protocols. Despite its accessibility and lower cost, TTE has limited sensitivity for LVT detection; therefore, CMR may be considered when clinical suspicion remains high despite a negative or inconclusive TTE. Although this study included a large, geographically diverse cohort of 3378 participants with structural heart disease and/or ventricular tachycardia, future research should address incomplete anticoagulation data and potential biases related to language and publication-date restrictions. Additionally, examining the impact of anticoagulation therapy and myocardial remodelling on LVT detection will further enhance the understanding and application of these diagnostic tools. Ultimately, optimizing LVT evaluation requires a tailored approach that balances the widespread feasibility of TTE with the definitive accuracy of CMR.

## Lead author biography


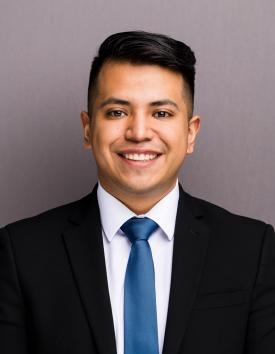


Ernesto Calderón-Martínez, MD, is a cardiovascular outcomes researcher currently at Texas Tech University Health Sciences Center El Paso. He has conducted clinical and translational research at the University of Texas Health Science Center at Houston focusing on heritable thoracic aortic disease (HTAD). His work integrates multicentre clinical cohorts, systematic reviews, and advanced epidemiologic methods to better understand cardiovascular risks. He has contributed to numerous peer-reviewed manuscripts and presentations at major international meetings. His broader academic interests include population health and the application of data-driven methods to improve risk stratification and clinical decision-making in diverse and underserved patient populations across healthcare settings.

## Supplementary Material

qyag150_Supplementary_Data

## Data Availability

All data and materials generated or analysed during this study are included in this published article.

## Protocol registration

The protocol for this review has been registered on PROSPERO databases under the following ID: CRD 42024558739.
