# Supplementary material for: Transthoracic echocardiography vs. cardiac magnetic resonance imaging for the diagnosis of left ventricular thrombus: an updated systematic review and diagnostic accuracy meta-analysis
Source: Eur Heart J Imaging Methods Pract. 2026 Sep 19;4(4):qyag150. doi: 10.1093/ehjimp/qyag150 (PMC13675715; doi:10.1093/ehjimp/qyag150)
Supplement: Supplementary file 1 [file qyag150_supplementary_data.docx]

**Supplementary Material**

**Supplementary Table 1. Pubmed**

| **Search** | **Results** |
| --- | --- |
| ((((Heart Ventricles[MeSH]) OR ("Heart Ventricle"[Title/Abstract]))  AND ((Thrombosis[MeSH]) OR  ("Thromboses"[Title/Abstract]) OR ("Thrombus"[Title/Abstract]))) OR  (((intracard*[Title/Abstract]) AND  (mass[Title/Abstract])) OR  ((ventric*[Title/Abstract]) OR (intracard*[Title/Abstract])) AND (Thromb*[Title/Abstract]) OR ("Left ventricular thrombus"[Title/Abstract]) OR ("LVT"[Title/Abstract]))  AND   ((Magnetic Resonance Imaging[MeSH]) OR  ("MRI"[Title/Abstract]) OR ("MR Imaging"[Title/Abstract]) OR ("Magnetic Resonance Imaging"[MeSH]) OR ("MRI"[Title/Abstract]) OR ("Cardiac MRI*"[Title/Abstract]) OR ("Cardiac Magnetic Resonance Imaging*"[Title/Abstract]) OR ("CMR"[Title/Abstract])) AND  ((Echocardiography[MeSH]) OR  ("Echocardiogram"[Title/Abstract]) OR ("Heart Ultrasound"[Title/Abstract]) OR ((Cardiac[Title/Abstract]) AND ("magnetic resonance"[Title/Abstract]) OR ("computed tomography"[Title/Abstract])) OR ((Transthoracic[Title/Abstract]) AND (echocardiograph*[Title/Abstract]) OR ("Echocardiogram"[Title/Abstract])) OR  ("TTE"[Title/Abstract]))) | 1161 |

**Supplementary Table 2. Scopus**

| **Search** | **Results** |
| --- | --- |
| ( TITLE-ABS-KEY ( heart  AND ventricles )  OR  TITLE-ABS-  KEY ( heart  AND ventricle )  OR  TITLE-ABS-KEY ( thrombosis )  OR  TITLE-ABSKEY ( thromboses )  OR  TITLE-ABS-KEY ( thrombus )  OR  TITLE-ABS-  KEY ( intracard* )  AND  TITLE-ABS-KEY ( mass )  OR  TITLE-ABSKEY ( ventric* )  OR  TITLE-ABS-KEY ( intracard* )  AND  TITLE-ABS- KEY ( thromb* )  OR  TITLE-ABS-  KEY ( left  AND ventricular  AND thrombus )  OR  TITLE-ABS-  KEY ( ¨lvt¨ )  AND  TITLE-ABS-  KEY ( magnetic  AND resonance  AND imaging )  OR  TITLE-ABSKEY ( ¨mri¨ )  OR  TITLE-ABS-KEY ( "MR Imaging" )  OR  TITLE-ABS-  KEY ( cardiac  AND mri* )  OR  TITLE-ABS-  KEY ( cardiac  AND magnetic  AND resonance  AND imaging )  OR  TITLE-ABS-  KEY ( ¨cmr¨ )  AND  TITLE-ABS-KEY ( echocardiography )  OR  TITLE-ABSKEY ( echocardiogram )  OR  TITLE-ABS-KEY ( heart  AND ultrasound )  OR  TITLEABS-KEY ( cardiac )  AND  TITLE-ABS-KEY ( magnetic  AND resonance )  OR  TITLEABS-KEY ( computed  AND tomography )  OR  TITLE-ABS-  KEY ( transthoracic  AND echocardiography )  OR  TITLE-ABS-KEY ( tte ) ) | 4845 |

**Supplementary Table 3. LILACS**

| **Search** | **Results** |
| --- | --- |
| (Heart Ventricules) AND (Thrombos*) OR (Left ventricular thrombus) OR (LVT) AND (Magnetic resonance Imaging*) OR (MRI) OR (MR Imaging) AND (Echocardiography ) OR (Echocardiogram)  OR (Heart Ultrasound) AND (Magnetic resonance)  OR (Computed tomography) | 7 |

**Supplementary Table 4. Clinical trials**

| **Search** | **Results** |
| --- | --- |
| Heart Ventricles OR Heart Ventricle AND Thrombosis OR Thromboses OR Thrombus OR intracar* AND mass OR ventric* OR intracard* AND Thromb* OR Left Ventricular Thrombus OR ¨LVT¨ \| Magnetic Resonance Imaging MRI OR cardiac MRI OR MRI OR MR imaging OR Cardiac Magnetic Resonance Imaging OR CMR AND  Echocardiography OR Echocardiogram OR Heart  Ultrasound OR cardiac MRI OR Computed Tomography OR Transrhoracic AND echocardiograph* OR echocardiogram OR TTE | 995 |

**Supplementary Table 5. Google Scholar**

| **Search** | **Results** |
| --- | --- |
| Google scholar:  "Heart Ventricle" AND "Thrombosis" OR  "Thrombus" OR (intracard* AND mass) OR  (ventric* AND Thromb*) OR "Left ventricular thrombus" AND "MRI" OR "Cardiac MRI" OR  "CMR" AND "Echocardiography" OR "Heart  Ultrasound" OR "TTE" | 100 |

**Supplementary Table 6. Cochrane**

| **ID** | **Search** | **Hits** |
| --- | --- | --- |
| #1 | MeSH descriptor: [Heart Ventricles] explode all trees | 2072 |
| #2 | (Heart Ventricle):ti,ab,kw | 14520 |
| #3 | MeSH descriptor: [Thrombosis] explode all trees | 7026 |
| #4 | (("Thromboses" OR "Thrombus")):ti,ab,kw | 3742 |
| #5 | ((intracard*) AND (mass)):ti,ab,kw | 27 |
| #6 | ((ventric* OR intracard*) AND (Thromb*)):ti,ab,kw | 3073 |
| #7 | (("Left ventricular thrombus" OR "LVT")):ti,ab,kw | 130 |
| #8 | MeSH descriptor: [Magnetic Resonance Imaging] explode all trees | 13664 |
| #9 | (("MRI" OR "MR Imaging" OR "Cardiac MRI*" OR "Cardiac Magnetic  Resonance Imaging*" OR "CMR")):ti,ab,kw | 43330 |
| #10 | MeSH descriptor: [Echocardiography] explode all trees | 5310 |
| #11 | (("Echocardiogram" OR "Heart Ultrasound" OR (Cardiac AND "magnetic resonance") OR "computed tomography" OR (Transthoracic AND echocardiograph*) OR "TTE")):ti,ab,kw | 26855 |
| #12 | #1 OR #2 | 15901 |
| #13 | #3 OR #4 | 9854 |
| #14 | #5 OR #6 OR #7 | 3128 |
| #15 | #8 OR #9 | 48452 |
| #16 | #10 OR #11 | 31327 |
| #17 | #12 AND #13 AND #14 AND #15 AND #16 | 29 |

**Supplementary Table 7. Web of Science**

| **Search** | **Results** |
| --- | --- |
| TS=((("Heart Ventricle" OR "Heart Ventricles") AND ("Thrombosis" OR "Thromboses" OR "Thrombus"))  OR  (((Thromb*) OR ("Left ventricular thrombus" OR "LVT")) AND ((ventric* OR intracard*) OR (intracard* AND mass))))  AND  TS=("Magnetic Resonance Imaging" OR "MRI" OR "MR Imaging" OR "Cardiac MRI*" OR "Cardiac Magnetic Resonance Imaging*" OR "CMR")  AND  TS=("Echocardiography" OR "Echocardiogram" OR "Heart Ultrasound" OR (Cardiac AND "magnetic resonance") OR "computed tomography" OR (Transthoracic AND echocardiograph*) OR "TTE") | 1342 |

**Supplementary Table 8. Embase**

| **Search** | **Results** |
| --- | --- |
| ('heart ventricle'/exp OR 'heart ventricle':ti,ab) AND ('thrombosis'/exp OR 'thrombosis':ti,ab OR 'thrombus':ti,ab) OR ((intracard*:ti,ab AND mass:ti,ab) OR (ventric*:ti,ab OR intracard*:ti,ab) AND thromb*:ti,ab OR 'left ventricular thrombus':ti,ab OR 'lvt':ti,ab) AND ('magnetic resonance imaging'/exp OR 'mri':ti,ab OR 'mr imaging':ti,ab OR 'cardiac mri*':ti,ab OR 'cardiac magnetic resonance imaging*':ti,ab OR 'cmr':ti,ab) AND ('echocardiography'/exp OR 'echocardiogram':ti,ab OR 'heart ultrasound':ti,ab OR (cardiac:ti,ab AND 'magnetic resonance':ti,ab) OR 'computed tomography':ti,ab OR (transthoracic:ti,ab AND echocardiograph*:ti,ab) OR 'tte':ti,ab) | 4826 |

**Supplementary Table 9. CINAHL**

| **Search** | **Results** |
| --- | --- |
| ((((Heart Ventricles) OR ("Heart Ventricle")) AND ((Thrombosis) OR ("Thromboses") OR  ("Thrombus"))) OR (((intracard*) AND (mass)) OR ((ventric*) OR (intracard*)) AND (Thromb*) OR ("Left ventricular thrombus") OR ("LVT")) AND ((Magnetic Resonance Imaging) OR ("MRI") OR ("MR Imaging") OR ("Magnetic Resonance Imaging") OR ("MRI") OR ("Cardiac MRI*") OR ("Cardiac Magnetic Resonance Imaging*") OR  ("CMR")) AND ((Echocardiography) OR ("Echocardiogram") OR ("Heart Ultrasound") OR ((Cardiac) AND ("magnetic resonance") OR ("computed tomography")) OR ((Transthoracic) AND (echocardiograph*) OR ("Echocardiogram")) OR ("TTE"))) | 843 |

Supplementary Table 10. Reasons for exclusion of articles in the full text stage

| Author | Title | Reason for exclusion |
| --- | --- | --- |
| Maini, Rohit | **Cardiac MRI as a useful tool to differentiate tumor and thrombus.** | Wrong study design |
| Goitein, O. | **The accuracy of cardiac MRI in differentiating between intra cardiac tumors and thrombi** | Wrong study design |
| Johnson, E.M. | Cardiac magnetic resonance **for the evaluation of suspected cardiac thrombus: Conventional and emerging techniques** | Wrong study design |
| Barbera, S. | **Echocardiographic recognition of left ventricular mural thrombus** | Wrong study design |
| Tasu, JP. | **Computed tomography and magnetic resonance imaging of** left ventricular thrombus | Wrong intervention |
| Krauser, DG | **Incremental diagnostic utility of contrast-enhanced MRI (CE-MRI) vs contrast echocardiography for detection of** left ventricular thrombus**: Morphologic predictors of improved thrombus detection by CE-MRI in patients with systolic dysfunction** | Wrong study design |

Supplementary Table 11. Characteristics of the cohorts and inclusion and exclusion criteria of the included studies

| **STUDY** | **COHORT** | **INCLUSION CRITERIA** | **EXCLUSION CRITERIA** |
| --- | --- | --- | --- |
| Phan J. et al  (2019) | STEMI patients treated at the tertiary center (Liverpool Hospital) with either PCI or thrombolytic therapy + PCI | Patients with Ischaemic symptoms lasting longer than 20 min, with ST- segment elevation in 2 or more contiguous leads on standard 12 lead ECG and a characteristic high sensitivity troponin T kinetic profile. | Significant chronic kidney disease  (eGFR b 30 ml/min/1.73 m2 or renal replacement   therapy), previous cardiac surgery, known cardiomyopathy, previous atrial fibrillation, significant psychiatric illness, age b18 years or N 85 years  and      contraindications    to      CMR (claustrophobia, gadolinium allergy and ferrous metallic implants). |
| Velangi P. et al  (2019) | Study patients were identified  from      the     University      of  Minnesota Cardiovascular  Magnetic Resonance Registry—including all patients who undergo CMR at the University of Minnesota. | Matched cohort design with LV thrombus patients matched to non- LV thrombus patients. | Patients with focal neurological deficits secondary to brain trauma, tumor, infection, or other nonvascular causes. |
| Karau B. et al (2025) | Patients aged 18 years and  above with new onset heart failure symptoms having a  reduced LVEF of 35% and less on 2D TEE at laboratory of AKUHN between January 2021 and December 202 | Patients were enrolled if 2D TEE and CMR were done within the 14-day interval. | Patients unable to undergo both imaging modalities within 14 days, having extreme claustropho-  bia, having an implanted MRI incompatible device, those  with risk of developing nephrogenic systemic fibrosis or worsening kidney dysfunction and having a previous diagnosis of LVT were excluded from the study design. |
| Barkhausen      J.  et al (2002) | Participants referred to the echocardiography laboratory  for evaluation of known or suspected intracardiac  thrombi | Participants referred to the echocardiography laboratory  for evaluation of known or suspected intracardiac  thrombi | NA |
| Beavers D. et al  (2021) | Patients with structural heart  disease           referred          for  radiofrequency ablation of recurrent VT | Patients with structural heart disease referred for VT ablation who were able to undergo all three imaging modalities (CMR, CT, TTE). | Patients unable to undergo all three imaging modalities were excluded, and 1 patient who underwent all three imaging modalities was excluded due to extensive device-related artifact. |
| Srichai M. et al  (2006) | Patients with IHD with a  history of remote MI >8 weeks. | Patients who had surgical and/or pathological evidence of the  presence or absence of LV thrombi. | NA |
| Weinsaft J. et al (2009) | Patients at high risk for LV thrombus due to recent myocardial infarction or  chronic heart failure. | Patients at high risk for LV thrombus due to recent myocardial infarction or chronic heart failure. | NA |
| Reindl M. et al  (2023) | STEMI patients enrolled in the  prospective ‘Magnetic Resonance Imaging in Acute ST-Elevation Myocardial  Infarction’ trial between 2011 | First-ever       STEMI       diagnosed  according to European Society of Cardiology/American College of Cardiology/American Heart  Association committee criteria and re-vascularized by primary PCI within 24h. | Inability or unwillingness to sign written informed consent, age <18 years, any history of a previous myocardial infarction or coronary intervention, Killip class ≥3 at time of CMR scan, an estimated glomerular filtration rate <30mL/min per 1.73m2, and any other specific contraindication  to       CMR      (pacemaker,       severe  claustrophobia, orbital foreign body, cerebral aneurysm clip, and known or suggested contrast allergy to  gadolinium. |
| Delewi R. et al  (2012) | Patients presenting with a first  STEMI | Patients presenting with a first STEMI who had had undergone  primary PCI with stent implantation within 12 h of symptom onset | Unsuccessful PCI, hemodynamic instability, elevation of CK or CKmyocardial band <10 times the local laboratory upper limit of normal, and contraindications for CMR. |
| Meurin P. et al  (2015) | High-risk population of patients with LV systolic dysfunction after anterior myocardial infarction | Patients with acute anterior wall ST  segment elevation myocardial infarction defined as follows were screened during hospitalization: characteristic symptoms of myocardial ischemia associated with persistent electrocardiographic ST elevation at the J point in 2 adjacent precordial leads (0.2 mV in leads V2 and V3 or 0.1 mV in the other precordial leads, V1 or V4V6) and subsequent release of biomarkers of myocardial necrosis. | CMR contraindication at baseline |
| Mollet N. et al  (2002) | Patients with Ischemic Heart Disease referred for MRI to assess myocardial viability | All consecutive patients with acute myocardial infarction (7 days’ duration), chronic myocardial infarction (7 days’ duration), or ischemic cardiomyopathy referred for MRI to assess myocardial  viability. | NA |
| Joshi S. et al  (2012) |  | All patients who had undergone  CMR       within     30     days      of  echocardiography were included in the study. | Patients undergoing coronary revascularization or cardiac resynchronization therapy between  echocardiography         and         CMR  examinations. |
| Staab W. et al  (2013) | Patients with a history of CAD | Patients who underwent TTE and CMR in routine follow up  examinations with history of CAD. | NA |
| Surder D. et al  (2015) | Patients with large, mainly anterior Acute Myocardial  Infarction. | Patients, mainly with anterior AMI, successfully reperfused by means of  primary PCI and with a left  ventricular ejection fraction (LVEF) less than 45%. All patients were studied with LGE-CMR at baseline. | Early dropouts and patients without CMR performed at baseline. |
| Weinsaft J. et al (2016) | Patients with acute MI enrolled in a prospective study focused on LV thrombus. | Patients were eligible for inclusión if admitted with acute ST-segment elevation MI (≥1.0 mm in at least 2 contiguous ECG leads). | Patients with contraindications to CMR (e.g., glomerular filtration rate <30 ml/min/1.73 m2, ferromagnetic  implants,      New       York       Heart  Association functional class IV) or taking warfarin (at the time of CMR) were excluded. |
| Weinsaft J. et al  (2011) | Consecutive patients at Duke University with LV systolic dysfunction. | The sole criterion for registry participation was impaired systolic  function,     defined     as    a     left  ventricular ejection fraction (LVEF) below 50% measured quantitatively on cine-CMR. | No patients were excluded based on clinical characteristics or other  criteria. |

*Abbreviations: STEMI, ST-segment Elevation Myocardial Infarction; PCI, Percutaneous coronary intervention; ECG, electrocardiogram; eGFR, estimated glomerular filtration rate; CMR, Cardiac Magnetic Resonance; LV, Left Ventricle; NA, Not Available; VT, Ventricular Tachycardia; CT, computed tomography; TTE, Transthoracic echocardiography; IHD, Ischemic Heart Disease; CK, creatine kinase; MRI, Magnetic Resonance Imaging; CAD, Coronary Artery Disease*

Supplementary Table 12. Subgroup Analysis of Transthoracic Echocardiography Sensitivity for Detecting Left Ventricular Thrombus

| **Variable** | **Subgroup** | **k** | **SMD (95% CI)** | **I² (%)** | **p-value (Between Groups)** |
| --- | --- | --- | --- | --- | --- |
| **Indication** | Post MI | 8 | 0.4608 (0.2881; 0.6434) | 69.6 |  |
|  | Left ventricular systolic dysfunction | 2 | 0.4000 (0.2615; 0.5565) | 8.9 |  |
|  | Known thrombous | 1 | 0.6667 (0.3334; 0.8889) | — |  |
|  | VT ablation | 1 | 0.2222 (0.0560; 0.5790) | — |  |
|  | ICD implantation | 1 | 0.1667 (0.0228; 0.6313) | — |  |
|  | CAD | 1 | 0.8140 (0.6699; 0.9041) | — |  |
|  | HF | 1 | 0.1026 (0.0521; 0.1919) | — | <0.0001 |
| **Study Design** | Prospective Cohort | 8 | 0.4706 (0.2534; 0.6996) | 82.8 |  |
|  | Retrospective Cohort | 6 | 0.3980 (0.2172; 0.6118) | 85.1 |  |
|  | RCT | 1 | 0.3529 (0.1679; 0.5959) | — | 0.7831 |
| **Contrast Echo** | No | 10 | 0.4595 (0.2609; 0.6718) | 87.0 |  |
|  | Yes | 5 | 0.3636 (0.2751; 0.4625) | 24.3 | 0.4220 |

Supplementary Table 13. Sensitivity Analysis of Transthoracic Echocardiography Sensitivity for Detecting Left Ventricular Thrombus

| **Author** | **Effect (95% CI: Lower; Upper)** | **I²** | **DFFITS** | **Cook's D** | **QE (del)** | **Is Influential** |
| --- | --- | --- | --- | --- | --- | --- |
| Omitting Phan J. et al, 2019 | -0.3151 (-0.9745; 0.3443) | 0.8212 | 0.0748 | 0.0061 | 72.7184 | no |
| Omitting Srichai M. et al, 2005 | -0.2296 (-0.8819; 0.4228) | 0.8103 | -0.2277 | 0.0552 | 68.5427 | no |
| Omitting Reindl M. et al, 2023 | -0.2547 (-0.9126; 0.4033) | 0.8251 | -0.1341 | 0.0197 | 74.3262 | no |
| Omitting Delewi R. et al, 2012 | -0.2261 (-0.8669; 0.4146) | 0.8228 | -0.2183 | 0.0492 | 73.3623 | no |
| Omitting Meurin P. et al, 2015 | -0.4798 (-1.0052; 0.0455) | 0.7952 | 0.5178 | 0.2385 | 63.4913 | no |
| Omitting Surder D. et al, 2015 | -0.3621 (-0.9988; 0.2745) | 0.8158 | 0.2419 | 0.0587 | 70.5799 | no |
| Omitting Weinsaft J. et al, 2016 | -0.2669 (-0.9223; 0.3885) | 0.8259 | -0.0878 | 0.0083 | 74.6840 | no |
| Omitting Weinsaft J. et al, 2011 | -0.2591 (-0.9156; 0.3974) | 0.8256 | -0.1161 | 0.0146 | 74.5535 | no |
| Omitting Chaosuwannakit N. et al, 2021 | -0.3135 (-0.9694; 0.3424) | 0.8232 | 0.0688 | 0.0051 | 73.5268 | no |
| Omitting Barkhausen J. et al, 2002 | -0.3571 (-0.9928; 0.2786) | 0.8185 | 0.2174 | 0.0476 | 71.6402 | no |
| Omitting Beavers D. et al, 2021 | -0.2313 (-0.8672; 0.4046) | 0.8240 | -0.1877 | 0.0360 | 73.8761 | no |
| Omitting Mollet N. et al, 2002 | -0.2875 (-0.9419; 0.3669) | 0.8257 | -0.0183 | 0.0004 | 74.5961 | no |
| Omitting Joshi S. et al, 2012 | -0.2253 (-0.8524; 0.4019) | 0.8237 | -0.1855 | 0.0347 | 73.7245 | no |
| Omitting Staab W. et al, 2013 | -0.4431 (-1.0242; 0.1379) | 0.7178 | 0.7503 | 0.3920 | 46.0587 | no |
| Omitting Karau B. et al, 2025 | -0.1371 (-0.7062; 0.4320) | 0.7564 | -0.6060 | 0.2648 | 53.3581 | no |

Supplementary Table 14. Subgroup Analysis of Transthoracic Echocardiography Specificity for Detecting Left Ventricular Thrombus

| **Variable** | **Subgroup** | **k** | **SMD (95% CI)** | **I² (%)** | **p-value (Between Groups)** |
| --- | --- | --- | --- | --- | --- |
| **Indication** | Post MI | 8 | 0.9843 (0.9644; 0.9931) | 58.7 |  |
|  | Left ventricular systolic dysfunction | 2 | 0.9587 (0.8623; 0.9885) | 88.3 |  |
|  | Known thrombous | 1 | 1.0000 (0.0000; 1.0000) | — |  |
|  | VT ablation | 1 | 1.0000 (0.0000; 1.0000) | — |  |
|  | ICD implantation | 1 | 1.0000 (0.0000; 1.0000) | — |  |
|  | CAD | 1 | 1.0000 (0.0000; 1.0000) | — |  |
|  | HF | 1 | 0.7865 (0.6893; 0.8595) | — | <0.0001 |
| **Study Design** | Prospective Cohort | 8 | 0.9827 (0.9371; 0.9954) | 87.5 |  |
|  | Retrospective Cohort | 6 | 0.9906 (0.9374; 0.9987) | 56.8 |  |
|  | RCT | 1 | 0.9837 (0.9507; 0.9947) | — | 0.8658 |
| **Contrast Echo** | No | 10 | 0.9847 (0.9479; 0.9956) | 78.5 |  |
|  | Yes | 5 | 0.9875 (0.9454; 0.9972) | 87.0 | 0.8378 |

Supplementary Table 15. Sensitivity Analysis of Transthoracic Echocardiography Specificity for Detecting Left Ventricular Thrombus

| **Author** | **Effect (95% CI: Lower; Upper)** | **I²** | **DFFITS** | **Cook's D** | **QE (del)** | **Is Influential** |
| --- | --- | --- | --- | --- | --- | --- |
| Omitting Phan J. et al, 2019 | 4.0298 (3.1285; 4.9310) | 0.8264 | 0.2835 | 0.0785 | 85.1502 | no |
| Omitting Srichai M. et al, 2005 | 4.3754 (3.2918; 5.4589) | 0.8184 | -0.1694 | 0.0319 | 87.7713 | no |
| Omitting Reindl M. et al, 2023 | 4.0277 (3.0743; 4.9810) | 0.7490 | 0.8097 | 0.5107 | 68.2447 | yes |
| Omitting Delewi R. et al, 2012 | 4.3479 (3.2592; 5.4367) | 0.8170 | -0.0925 | 0.0094 | 87.0129 | no |
| Omitting Meurin P. et al, 2015 | 4.2655 (3.2146; 5.3163) | 0.8219 | 0.0317 | 0.0010 | 88.4445 | no |
| Omitting Surder D. et al, 2015 | 4.3668 (3.2867; 5.4469) | 0.8237 | -0.1658 | 0.0300 | 89.3206 | no |
| Omitting Weinsaft J. et al, 2016 | 4.2900 (3.2075; 5.3725) | 0.8107 | 0.0568 | 0.0034 | 84.6433 | no |
| Omitting Weinsaft J. et al, 2011 | 4.4207 (3.4018; 5.4395) | 0.8172 | -0.3983 | 0.1556 | 84.4389 | no |
| Omitting Chaosuwannakit N. et al, 2021 | 4.2869 (3.2048; 5.3689) | 0.8098 | 0.0660 | 0.0046 | 84.3689 | no |
| Omitting Barkhausen J. et al, 2002 | 4.1902 (3.2200; 5.1604) | 0.8264 | -0.0540 | 0.0030 | 89.8898 | no |
| Omitting Beavers D. et al, 2021 | 4.0523 (3.1384; 4.9661) | 0.8264 | 0.2470 | 0.0600 | 85.8743 | no |
| Omitting Mollet N. et al, 2002 | 4.3895 (3.3316; 5.4475) | 0.8264 | -0.2698 | 0.0764 | 90.0534 | no |
| Omitting Joshi S. et al, 2012 | 4.1375 (3.1831; 5.0918) | 0.8264 | 0.0832 | 0.0070 | 88.5573 | no |
| Omitting Staab W. et al, 2013 | 4.0633 (3.1435; 4.9831) | 0.8264 | 0.2282 | 0.0514 | 86.2305 | no |
| Omitting Karau B. et al, 2025 | 4.3999 (3.5219; 5.2778) | 0.6542 | -0.5840 | 0.2091 | 48.2789 | no |

Supplementary Table 16. Subgroup Analysis of Transthoracic Echocardiography Specificity for Detecting Left Ventricular Thrombus compared to Cine-CMR with LGE-CMR as gold standard

| **Variable** | **Subgroup** | **k** | **SMD (95% CI)** | **I² (%)** | **p-value (Between Groups)** |
| --- | --- | --- | --- | --- | --- |
| Study design | Retrospective Cohort | 3 | 0.9592 [0.9093; 0.9822] | 82.7 |  |
|  | RCT | 1 | 0.9837 [0.9507; 0.9947] | -- |  |
|  | Prospective Cohort | 1 | 0.9333 [0.8127; 0.9783] | -- |  |
|  |  |  |  |  | 0.2005 |
| Indication | Post MI | 4 | 0.9728 [0.9567; 0.9830] | 37.5 |  |
|  | Left ventricular systolic dysfunction | 1 | 0.9087 [0.8627; 0.9403] | -- |  |
|  |  |  |  |  | 0.0002 |
| Contrast Echo | No | 3 | 0.9686 [0.9485; 0.9810] | 39.1 |  |
|  | Yes | 2 | 0.9579 [0.8629; 0.9880] | 87.9 |  |
|  |  |  |  |  | 0.6686 |

Supplementary Table 17. Sensitivity Analysis of Transthoracic Echocardiography Specificity for Detecting Left Ventricular Thrombus compared to Cine-CMR with LGE-CMR as gold standard

| **Author** | **Effect (95% CI: Lower; Upper)** | **I^2** | **DFFITS** | **Cook's D** | **QE (del)** | **Is Influential** |
| --- | --- | --- | --- | --- | --- | --- |
| Srichai M. et al, 2005 | 3.045 (2.672; 3.417) | 0.7968 | 0.7575 | 0.5739 | 14.77 | yes |
| Weinsaft J. et al, 2016 | 2.938 (2.598; 3.278) | 0.7413 | 0.6695 | 0.4482 | 11.60 | no |
| Weinsaft J. et al, 2011 | 3.576 (3.107; 4.044) | 0.3745 | -3.5064 | 12.2948 | 4.80 | yes |
| Mollet N. et al, 2002 | 3.129 (2.791; 3.468) | 0.8171 | -0.1125 | 0.0127 | 16.40 | no |
| Chaosuwannakit N. et al, 2021 | 2.929 (2.589; 3.269) | 0.7352 | 0.6875 | 0.4726 | 11.33 | yes |

Supplementary Table 18. Subgroup Analysis of CineCMR Sensitivity for Detecting Left Ventricular Thrombus compared to TTE with LGE-CMR as gold standard

| **Variable** | **Subgroup** | **k** | **SMD (95% CI)** | **I² (%)** | **p-value (Between Groups)** |
| --- | --- | --- | --- | --- | --- |
| Study design | Retrospective Cohort | 3 | 0.7620 [0.5911; 0.8765] | 74.4 |  |
|  | RCT | 1 | 0.6429 [0.3763; 0.8430] | -- |  |
|  | Prospective Cohort | 1 | 0.5000 [0.2439; 0.7561] | -- |  |
|  |  |  |  |  | 0.2458 |
| Indication | Post MI | 4 | 0.7362 [0.5655; 0.8568] | 66.5 |  |
|  | Left ventricular systolic dysfunction | 1 | 0.5833 [0.3834; 0.7591] | -- |  |
|  |  |  |  |  | 0.2247 |
| Contrast Echo | No | 3 | 0.7563 [0.5533; 0.8861] | 73.9 |  |
|  | Yes | 2 | 0.6053 [0.4445; 0.7461] | 0.0 |  |
|  |  |  |  |  | 0.2195 |

Supplementary Table 19. Sensitivity Analysis of CineCMR Sensitivity for Detecting Left Ventricular Thrombus compared to TTE with LGE-CMR as gold standard

| **Author** | **Effect (95% CI: Lower; Upper)** | **I^2** | **DFFITS** | **Cook's D** | **QE (del)** | **Is Influential** |
| --- | --- | --- | --- | --- | --- | --- |
| Srichai M. et al, 2005 | 0.495 (-0.003; 0.992) | 0.0000 | 2.2631 | 5.1218 | 2.00 | yes |
| Weinsaft J. et al, 2016 | 1.133 (0.714; 1.551) | 0.7369 | -0.2824 | 0.0798 | 11.40 | no |
| Weinsaft J. et al, 2011 | 1.265 (0.812; 1.718) | 0.6649 | -1.0034 | 1.0069 | 8.95 | yes |
| Mollet N. et al, 2002 | 1.200 (0.778; 1.623) | 0.6584 | -0.6855 | 0.4699 | 8.78 | yes |
| Chaosuwannakit N. et al, 2021 | 1.065 (0.650; 1.480) | 0.7460 | 0.1084 | 0.0118 | 11.81 | no |

Supplementary Figure 2. Funnel plot of DOR for TTE

Supplementary Figure 2. Funnel plot of DOR for CMR
